# Human meibomian gland organoids to study epithelial homeostasis and dysfunction

**DOI:** 10.1093/procel/pwaf095

**Published:** 2025-11-09

**Authors:** Chuyue Yu, Xichen Wan, Jinsong Wei, Zhaoting Xu, Xingru Wu, Xiaoye Wang, Yabo Mi, Yiming Zhang, Dan Wu, Xujiao Zhou, Qihua Le, Jianjiang Xu, Chen Zhao, Xinghuai Sun, Xingtao Zhou, Jiaxu Hong, Bing Zhao

**Affiliations:** School of Basic Medical Sciences, Institute of Biomedical Innovation, The First Affiliated Hospital, Jiangxi Medical College, Nanchang University, Nanchang 330031, China; Department of Ophthalmology, Eye & ENT Hospital, State Key Laboratory of Brain Function and Disorders, Fudan University, Shanghai 200031, China; Department of Ophthalmology, Eye & ENT Hospital, State Key Laboratory of Brain Function and Disorders, Fudan University, Shanghai 200031, China; School of Basic Medical Sciences, Institute of Biomedical Innovation, The First Affiliated Hospital, Jiangxi Medical College, Nanchang University, Nanchang 330031, China; School of Basic Medical Sciences, Institute of Biomedical Innovation, The First Affiliated Hospital, Jiangxi Medical College, Nanchang University, Nanchang 330031, China; School of Basic Medical Sciences, Institute of Biomedical Innovation, The First Affiliated Hospital, Jiangxi Medical College, Nanchang University, Nanchang 330031, China; School of Basic Medical Sciences, Institute of Biomedical Innovation, The First Affiliated Hospital, Jiangxi Medical College, Nanchang University, Nanchang 330031, China; School of Basic Medical Sciences, Institute of Biomedical Innovation, The First Affiliated Hospital, Jiangxi Medical College, Nanchang University, Nanchang 330031, China; School of Basic Medical Sciences, Institute of Biomedical Innovation, The First Affiliated Hospital, Jiangxi Medical College, Nanchang University, Nanchang 330031, China; Department of Facial Plastic and Reconstructive Surgery, Eye & ENT Hospital of Fudan University, Shanghai 200031, China; Department of Ophthalmology, Eye & ENT Hospital, State Key Laboratory of Brain Function and Disorders, Fudan University, Shanghai 200031, China; Department of Ophthalmology, Eye & ENT Hospital, State Key Laboratory of Brain Function and Disorders, Fudan University, Shanghai 200031, China; Department of Ophthalmology, Eye & ENT Hospital, State Key Laboratory of Brain Function and Disorders, Fudan University, Shanghai 200031, China; Department of Ophthalmology, Eye & ENT Hospital, State Key Laboratory of Brain Function and Disorders, Fudan University, Shanghai 200031, China; Department of Ophthalmology, Eye & ENT Hospital, State Key Laboratory of Brain Function and Disorders, Fudan University, Shanghai 200031, China; Department of Ophthalmology, Eye & ENT Hospital, State Key Laboratory of Brain Function and Disorders, Fudan University, Shanghai 200031, China; Department of Ophthalmology, Eye & ENT Hospital, State Key Laboratory of Brain Function and Disorders, Fudan University, Shanghai 200031, China; NHC Key Laboratory of Myopia and Related Eye Diseases, Shanghai 200031, China; Shanghai Key Laboratory of Rare Disease Gene Editing and Cell Therapy, Shanghai Engineering Research Center of Synthetic Immunology, Shanghai 200032, China; Department of Ophthalmology, Children’s Hospital of Fudan University, National Pediatric Medical Center of China, Shanghai 201102, China; School of Basic Medical Sciences, Institute of Biomedical Innovation, The First Affiliated Hospital, Jiangxi Medical College, Nanchang University, Nanchang 330031, China; Z Lab, bioGenous BIOTECH, Shanghai 200438, China

**Keywords:** meibomian gland, organoids, tissue stem cells, FGF10, nicotinamide, MAPK signaling, single-cell atlas, orthotopic transplantation

## Abstract

Meibomian glands (MGs) are holocrine glands that secrete lipids to maintain the homeostasis of the ocular surface, and their dysfunction leads to dry eye disease. Herein, we established long-term 3D organoid culture for murine and human MGs, which retained the cell lineages and lipid-producing ability. The organoids mimicked the drug treatment responses and generated functional MGs after orthotopic transplantation. Inspired by organoid cultures, we found FGF10 eye drops could rescue all-trans retinoic acid-induced MG dysfunction in mice. Besides, nicotinamide uniquely hampered the human MG organoid expansion by inhibiting FGF10 signaling. Single-cell atlas and lipidome not only aligned the delineated cell types and featured lipids between human MGs and organoids, but also highlighted MAPK signaling inhibition enhanced acinar cell differentiation and functional maturation of MG organoids. In summary, this study established an organoid platform to explore epithelial homeostasis and dysfunction of MGs, facilitating drug development and regenerative medicine for dry eye disease.

## Introduction

Meibomian glands (MGs), the holocrine sebaceous glands (SGs) located in the eyelids, are essential for the homeostasis of the tear film and ocular surface. MGs are characterized by a central duct surrounded by acini that are responsible for lipid production. Under homeostasis, the basal cells in acini could self-renew and differentiate to acinar cells, which are also called meibocytes. The meibocytes begin to synthesize lipid while migrating toward the center of the acini. Finally, the meibocytes rupture to release the lipid content. Delivered by the central duct to the eyelid margin, the secreted lipid forms the outermost layer of the preocular tear film to prevent tear evaporation and environmental insults (Mathers, 2004).

Meibomian gland dysfunction (MGD) is the leading cause of evaporative dry eye disease (Hwang et al., 2017), which is featured by poor vision quality, eye irritation, ocular surface inflammation, and discomfort, affecting 38%–68% population worldwide. However, the only treatment for MGD is palliative care (Sun et al., 2020). Various risky factors were reported, including aging, drug treatment, and congenital defect (Bu et al., 2019, 2021; Caffery and Josephson, 1988; Hom et al., 1990; Liu et al., 2016; Nien et al., 2011; Sullivan et al., 2000, 2002), while the underlying pathology of MGD would be the stem cell exhaustion. To this end, the culture of MG stem cells, which could self-renew and differentiate into functional meibocytes *in vitro*, would be promising for MGD therapeutics. However, existing 2D immortalized MG epithelial cells are difficult to simulate in *in vivo* responses (Liu et al., 2010; Xie et al., 2018). Thus, it is urgent to create an *ex vivo* model for exploring the homeostasis and dysfunction of MGs.

Organoids with three-dimensional (3D) structures derived from adult stem cells accurately mimic physiological aspects of parental tissues *ex vivo*. Thus, organoids are used to model organ development and disease progression (Clevers, 2016). However, no approach has been established to generate human MG organoids (hMGOs) yet, limiting the study of the physiology and pathology of MGs. In this study, we achieved long-term expansion of murine and human MGs as 3D organoids, which recapitulated the characteristics of tissues, and modeled the MG degenerative diseases induced by chemical injuries *ex vivo*. Notably, hMGOs could engraft and undergo functional maturation after orthotopic transplantation into mice. Furthermore, we probed the cell types and lipidome of parallel human MG tissues and organoids using scRNA-seq and lipidomic analysis, highlighting that the MAPK signaling is critical for meibocyte maturation. Taken together, this study described an organoid platform to explore the homeostasis and diseases of MGs and provided a useful platform for drug testing and regenerative medicine.

## Results

### Establishment of functional murine MG organoids

**Figure 1.**
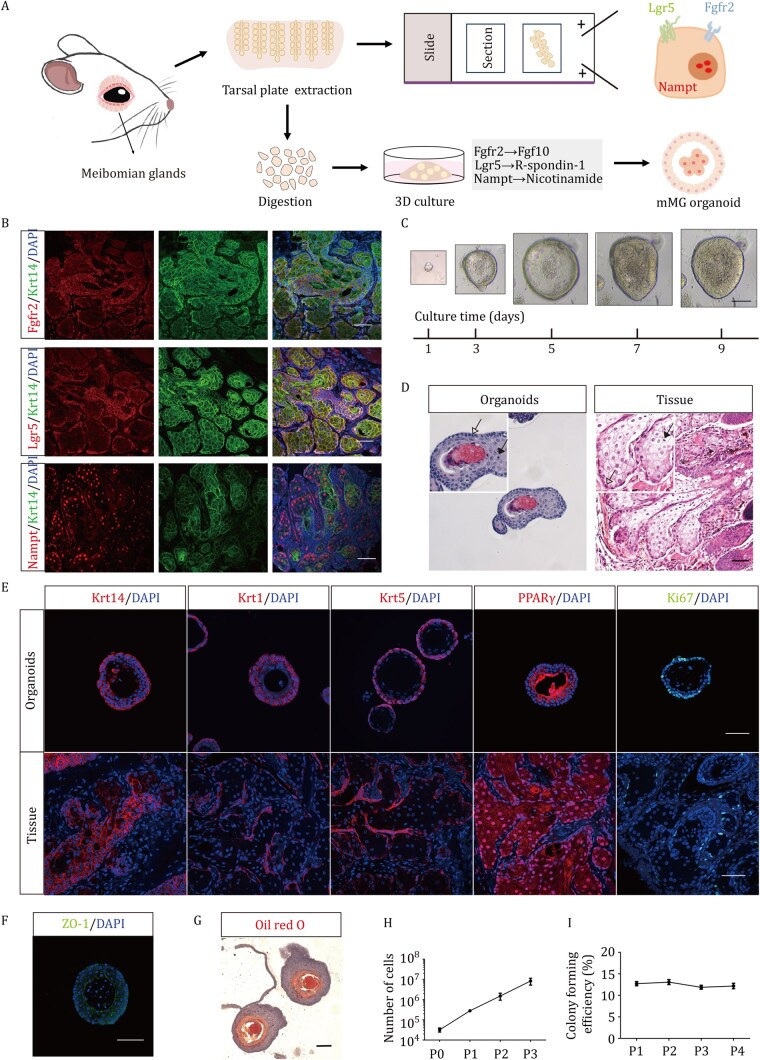
**Generation of functional murine MG organoids (mMGOs)**. (A) Schematic diagram of organoid culture from murine MG tissue. (B) Immunofluorescent staining for Fgfr2, Lgr5, and Nampt in murine MG tissue (marked by Krt14). Scale bar, 50 µm. (C) Representative bright-field images of the outgrowth of a single organoid during the 9-day culture period. Scale bar, 100 µm. (D) H&E staining of passage 5, Day 9 organoid, and the murine MG tissue. White box indicates regions shown in higher magnifications (×2). Filled arrow indicates basal cells, and empty arrow indicates meibocytes with a pyknotic nucleus. Scale bar, 50 µm. (E) Immunofluorescent staining for the MG epithelial cell marker (Krt14), ductal cell marker (Krt1), basal cell marker (Krt5), meibocyte marker (PPARγ), and the proliferation marker Ki67 in organoids and in tissue. Scale bar, 50 µm. (F) Immunofluorescent staining for ZO-1 in mMGOs. Scale bar, 50 µm. (G) Oil Red O staining of lipids in mMGOs. Scale bar, 50 µm. (H and I) The cell number (I) and colony forming efficiency (J) of mMGOs during passage 0–4. See also Figs. S1 and S2.

Before formulating the culture protocol for meibomian gland organoids (MGOs), we first examined the key signaling pathways in the MG tissues (Fig. 1A). FGF signaling is essential for mammary gland development, and loss of Fgf10 or its receptor Fgfr2 led to placode defects during embryogenesis (Kim et al., 2013; Mailleux et al., 2002). The Wnt signaling is the key driver of adult stem cells in most tissues. As the receptor for R-spondins, Lgr5 marks adult stem cells in actively self-renewing organs (Nusse and Clevers, 2017). In addition to the extracellular signals, nicotinamide phosphoribosyltransferase (Nampt) is a key enzyme that regulates the intracellular nicotinamide adenine dinucleotide (NAD) pool, which is crucial for the cellular redox stage (Garten et al., 2015). Immunostaining showed that Fgfr2, Lgr5, and Nampt were highly expressed in MG tissues, which were marked by Krt14 (Fig. 1B). Therefore, we supplemented FGF10, R-spondin-1, and nicotinamide as the core factors in murine MG organoid (mMGO) culture medium. During 9 days’ incubation, the seeded MG cell gradually formed the spheroid structure, and became solid and opaque during expansion (Figs. 1C and S1A). H&E staining showed organoids and tissues shared similar architecture: the outer layer of the organoid was lined with small and compact cells, which resembled the basal cells. While the internal cells displayed lower nucleocytoplasmic ratios and condensed cell nuclei ruptures, which resembled the differentiated acinar cells (Fig. 1D).

To further confirm the cell lineages in organoids, we stained the typical cell markers. In the MGs, the basal acinar cells (Krt5^+^) retained the ability of self-renewal, and moved toward the center of the acini to form terminal differentiated gland acinar cells/meibocytes (PPARγ^+^). Ideally, the organoids well preserved the cell lineages and their niche in MGs, exhibiting proliferative Ki67^+^ or Krt5^+^ basal acinar cells lining the out layers, and PPARγ^+^ meibocytes locating in the inner layers (Fig. 1E). Besides, the inner cell also gained cell polarity, with the apical side facing inside (Fig. 1F). Importantly, the mMGOs could be long-term expanded up to 20 passages (Figs. 1H, 1I and S1D).

The main function of MG is meibum/lipid secretion. Oil Red O staining of MGOs showed that inner layer cells were Oil Red O positive, and the inner cavity displayed much condensed staining (Figs. 1G and S1B), indicating the meibocytes secreted lipid into the cavity. To further validate the recapitulation of the holocrine secretion mode in our MGOs, we performed neutral lipid staining and transmission electron microscopy (TEM). Neutral lipid staining revealed specific accumulation of lipid droplets in the organoid center (Fig. S2A), confirming the inward migration and maturation of meibocytes. Crucially, TEM imaging definitively captured the entire holocrine process, it visualized the progression from basal cells (Layer 1) with prominent nuclei, dense heterochromatin, and scarce lipids; through differentiating meibocytes (Layer 2) featuring dramatic lipid accumulation and honeycomb-like structures; to fully mature, anucleate cells (Layers 3–4) filled with lipids ready for release into the inner cavity (Fig. S2B).

To further characterize the transcriptomic similarity between MGs and MGOs, we performed bulk RNA-seq and set the irrelevant lacrimal gland as controls. The organoids displayed a similar gene expression pattern with the MGs (Fig. S1E and S1F), and showed a significant enrichment of MG signatures: *Krt6*, *Lrig1*, and lipid synthesis-related genes *ElovI6*, *Scd1*, *Far1*, and *Hmgcs1* (Fig. S1G). Besides, we found that age and sex do not affect the MGO establishment (Fig. S1C). In summary, the long-term expandable and functional MGOs preserved the cell lineages, gene expression signatures, and lipid-secretion capability of MGs.

### FGF10 sustained MG organoids’ expansion and prevented MG dysfunction

**Figure 2.**
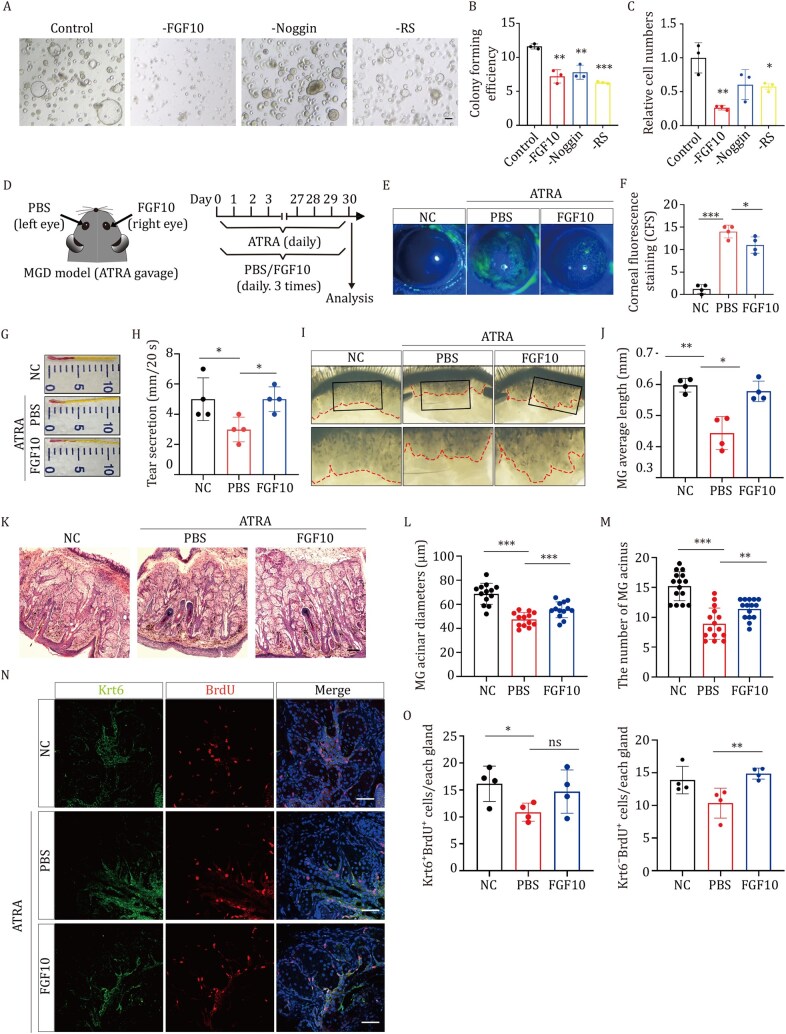
**FGF10 is required for the organoid expansion and prevents MG dysfunction (MGD)**. (A) Representative bright-field images of the mMGOs in 4 conditions (removing the FGF10, Noggin, Rspo-1, and control). Scale bar, 100 µm. (B and C) Quantification of colony-forming efficiency (B) and cell numbers (C) of organoids in the four indicated conditions. Data were represented as mean ± SEM in 3 independent experiments. Unpaired two-tailed Student’s *t*-test: *P < 0.05, **P < 0.01, ***P < 0.001. (D) Schematic of the workflow and the timeline for the ATRA-induced MGD model and FGF10 eye drops administration. (E and F) Corneal fluorescein staining (E) images and (F) scores of normal control mice and ATRA-induced MGD mice treated with PBS or FGF10 eye drops. NC: normal control group without any treatment; PBS: ATRA-induced MGD group treated with PBS eye drops; FGF10: ATRA-induced MGD group treated with FGF10 eye drops. Data were represented as mean ± SEM. *n* = 4 mice for each group. Unpaired two-tailed Student’s *t*-test: *P < 0.05, ***P < 0.001. (G and H) Representative images (G) and measurement (H) of tear secretion volume using the phenol red thread test of the indicated groups. Data were represented as mean ± SEM. *n* = 4 mice for each group. Unpaired two-tailed Student’s *t*-test: *P < 0.05. (I and J) Representative bright-field images of MGs (I) and quantification of MG length (J) of the indicated groups. Red dotted line indicates the margin of MGs. Lower panel: higher magnifications of the upper panel. Data were represented as mean ± SEM. *n* = 4 mice for each group. Unpaired two-tailed Student’s *t*-test: *P < 0.05, **P < 0.01. (K–M) H&E staining (K) and quantification of size (L) and number (M) of MG acini of the indicated groups. Quantified data were determined in 12 randomly selected fields. Data were represented as mean ± SEM. Unpaired two-tailed Student’s *t*-test: **P < 0.01, ***P < 0.001. Scale bar, 100µm. (N) Immunofluorescent staining for Krt6 (ductal cell marker) and BrdU (proliferation marker) of the indicated groups. Scale bar, 50µm. (O) Quantification of the number of proliferative non-ductal cells (Krt6^−^/BrdU^+^ cells) and ductal cells (Krt6^+^/BrdU^+^ cells) in the indicated groups. Data were represented as mean ± SEM. *n* = 4 mice for each group. Unpaired two-tailed Student’s *t*-test: *P < 0.05, **P < 0.01. See also Figs. S3 and S4.

The regeneration of MG depends on the self-renewal acinar basal cells, and the long-term expandable MGO culture system highlighted critical signaling pathways that boost basal cell amplification (Fig. 1E). We reasoned that the dominating signaling could be identified using an organoid culture system. After removing FGF10, Noggin, and R-spondin1 from culture medium individually, we found that MGOs were most sensitive to FGF10, rather than Noggin or R-spondin1 (Fig. 2A–C). FGF10 was a critical factor in regulating the development of the eyelid and the proliferation of immortalized human MG epithelial cells (Nuwormegbe et al., 2022; Tao et al., 2005), while its role in regeneration was less investigated. Inspired by the above, we then determined the function of FGF10 in MG dysfunction (MGD).

To establish a mouse MGD model, we optimized the previously described retinoic acid method by replacing 13-*cis* retinoic acid (13-cis-RA) with all-*trans* retinoic acid (ATRA) (Ibrahim and Elwan, 2017), since it is generally accepted that the effects of 13-cis-RA are mediated by isomerization to the more transcriptionally active ATRA form (Armstrong et al., 2005). Mice were treated with ATRA through gavage for a month to induce MG damage, and the FGF10 solution was dripped into the right eye (phosphate buffered saline, PBS for the left eye) three times/day as the therapeutic group (Fig. 2D). Ophthalmologic examinations, including ocular epithelial integrity, tear secretion and MG length, were conducted to score the MGD development and FGF10 treatment. Compared with the untreated group, the ATRA + PBS group showed pervaded corneal fluorescein staining (CFS), indicating compromised ocular epithelium. In addition, tear secretion ability and MG length were also reduced in the ATRA + PBS group (Fig. 2E–J). Histologically, ATRA + PBS also reduced the number and size of the acini in the MGs (Fig. 2K–M) and inhibited the proliferative cells in both the ductal (Krt6^+^ cells) and non-ductal cells (Krt6^−^ cells) of the MGs (Figs. 2N, 2O, S3A and S3B). Although no significant effects were found in the expression of PPARγ, the lipid synthesis (oil-red) was inhibited in the ATRA + PBS group (Fig. S3C and S3D). The above data suggested that the ATRA-induced MGD model was successfully established. Interestingly, based on the MGD model, the ATRA + FGF10 group exhibited significant MG recovery in all aspects, compared with the ATRA + PBS group. Ideally, ATRA + FGF10 rescued ocular epithelium integrity, tear secretion, gland architecture, cell proliferation, and lipid synthesis.

To dive deeper into the role of FGF10 signaling during MGD, we performed phospho-p38 (downstream indicator of FGF10 signaling) and KRT14 co-immunostaining, and found that ATRA treatment reduced the phospho-p38 signal and FGF10 application restores it (Fig. S4). These data demonstrated that FGF10 contributed to the MGs homeostasis, and the activation of FGF10 signaling alleviated the progression of MGD.

### MG organoids mimicked the drug responses

Since the MGOs could recapitulate the physiological aspects of MGs, we reasoned that the MGOs might be used to discover the drugs that resolved dry eye disease. To demonstrate this, we selected drugs with known effects on MGs: ATRA and 13-cis-RA were confirmed to have a detrimental effect, resulting in cell death, atrophy, and altered gene expression (Samarawickrama et al., 2015). Azithromycin (AZM) can stimulate lipid synthesis in iHMGECs and is the prescription medication for the treatment of MGD (Liu et al., 2014; Tao and Tao, 2020). Rapamycin, the mTOR signaling pathway inhibitor, is a potential drug for the treatment of age-related disease (Selvarani et al., 2021). Dihydrotestosterone (DHT) is a major androgen that has been considered to regulate MG function by suppressing inflammation (Sahin et al., 2020) and enhancing lipid synthesis (Ibrahim and Elwan, 2017).

**Figure 3.**
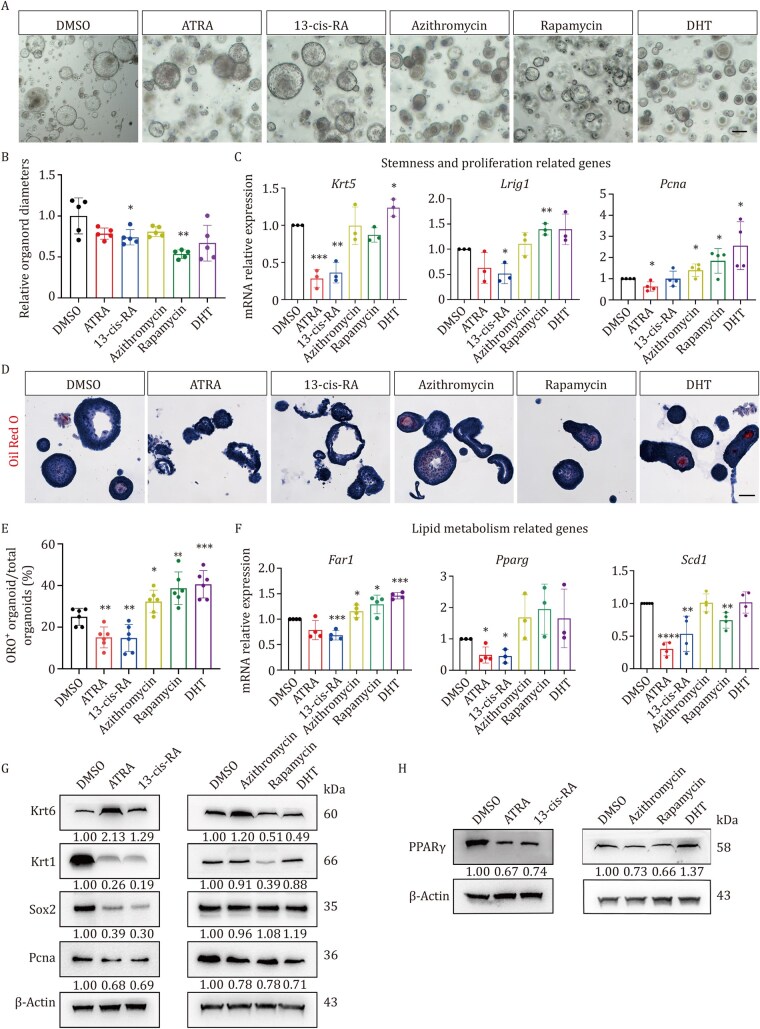
**Organoids mimic the drug response of MGs**. (A) Representative bright-field images of MGOs incubated with 0.1% DMSO, 100 µmol/L ATRA, 100 µmol/L 13-cis RA, 10 µmol/L AZM, 10 µmol/L Rapamycin, and 10 µmol/L DHT for 3 days, respectively. Scale bar, 100 µm. (B) Quantification of the diameters of organoids of the indicated treatment. Quantified data were determined in five randomly selected fields. Data were represented as mean ± SEM. Unpaired two-tailed Student’s *t*-test: *P < 0.05, **P < 0.01. (C) Quantitative real-time PCR (qRT-PCR) analysis of the expression level of proliferative and stemness-related genes, including *Krt-5*, *Lrig1*, and *Pcna*, of the organoids after indicated treatment. Data are represented as mean ± SEM in ≥3 independent experiments. Unpaired two-tailed Student’s *t*-test: *P < 0.05, **P < 0.01, ***P < 0.001. (D) Oil Red O staining of lipids in organoids with indicated treatment. Scale bar, 50 µm. (E) Quantification of the number of Oil Red O^+^ organoids divided by the total number of organoids with the indicated treatment. Quantified data were determined in six randomly selected fields. Data are represented as mean ± SEM. Unpaired two-tailed Student’s *t*-test: *P < 0.05, **P < 0.01, ***P < 0.001. (F) Quantitative real-time (RT)-PCR analysis of the expression level of lipid metabolism-related genes, including *Far*, *Pparg*, and *Scd1*, with RNA extracted from the organoids after indicated treatment. Data were represented as mean ± SEM in ≥3 independent experiments. Unpaired two-tailed Student’s *t*-test: *P < 0.05, **P < 0.01, ***P < 0.001. (G and H) Western blot analysis of Krt6, Krt1, Sox2, Pcna (stemness-related protein) (G), and PPARγ (metabolism-related protein) (H) protein expression in organoids with the indicated treatment. β-Actin served as a loading control. The relative band intensity that was normalized to the loading control was marked under each band. *n* = 3 biological replicates. See also Fig. S5.

We exposed the MGOs to those drugs individually and followed by the morphologic and functional characterizations. Compared to the dimethyl sulfoxide (DMSO) group, treatment with 100 µmol/L 13-cis RA, 10 µmol/L Rapamycin, and 10 µmol/L DHT resulted in a significant decrease in organoid size (Fig. 3A and 3B). The inner cavity of organoids became condensed after being treated with 10 µmol/L AZM and 10 µmol/L DHT, suggesting that AZM and DHT might induce the differentiation (Fig. 3A). Administration of 100 µmol/L ATRA and 100 µmol/L 13-cis-RA significantly inhibited the expression of proliferation and stemness genes (Xie et al., 2018), including *Krt5*, *Lrig1*, and *Pcna*, as well as the protein level of PCNA and Sox2 (Fig. 3C and 3G). Furthermore, the MG markers, *Krt10* and *Krt14*, decreased after the treatment with ATRA and 13-cis-RA, while the ductal cells marker gene *Krt6* was upregulated. The data suggested that the effects of the ATRA and 13-cis RA on the acinar and duct cells of the MGs might be different (Figs. 3G, S5A and S5B).

Regarding lipid synthesis, AZM, Rapamycin, and DHT treatments resulted in a significant increase in lipid production capacity, while ATRA and 13-cis RA led to a decrease, as indicated by the Oil Red O staining (Fig. 3D and 3E). Additionally, ATRA and 13-cis RA led to the reduction of lipid synthesis-related genes *Far1*, *Pparg*, and *Scd1* while promoting fatty acid synthase (*Fasn*) and Perilipin (*Plin2*) expression, suggesting that ATRA and 13-cis RA affected specific stages of differentiation (Figs. 3F,  S5C and S5D). In summary, the MGOs could rapidly assess the drug effects and precisely simulate drug responses *ex vivo*.

### Establishment of human MG organoids for orthotopic transplantation

**Figure 4.**
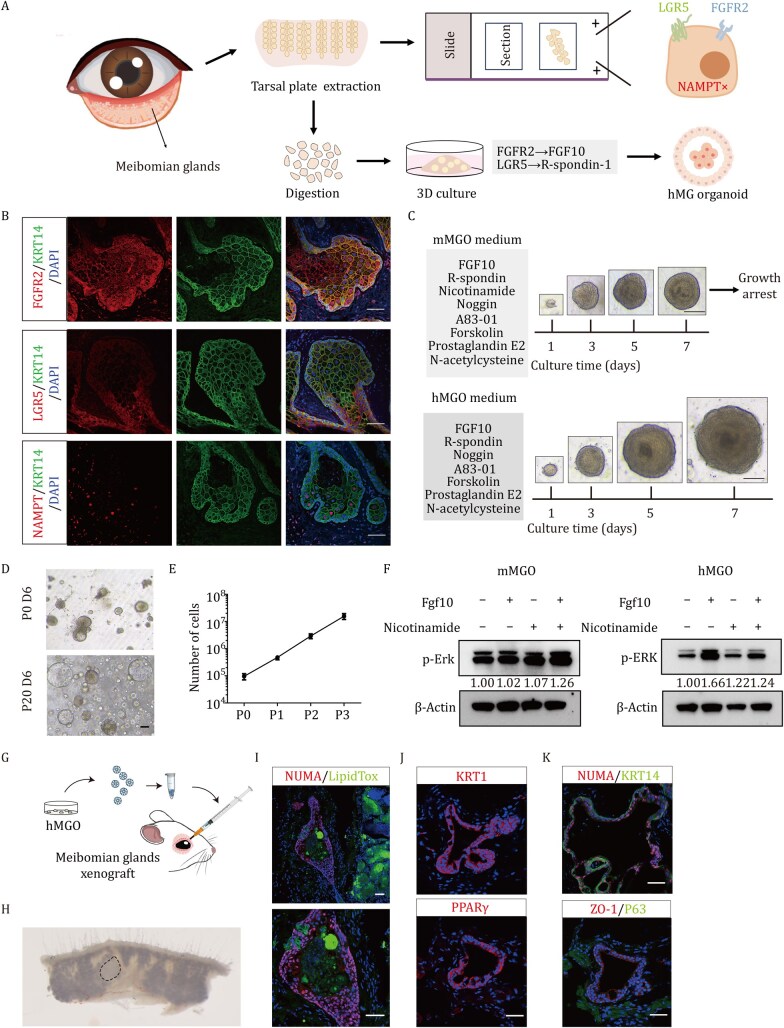
**Establishment of human MG organoids (hMGOs) for orthotopic transplantation**. (A) Schematic diagram of organoid culture from human MG tissue. (B) Immunofluorescent staining for FGFR2, LGR5, and NAMPT in human MG tissue (marked by KRT14). Scale bar, 50 µm. (C) Representative bright-field image of hMGOs with mMGO medium and hMGO medium at the indicated days. Scale bar, 50 µm. (D) Representative images of hMGOs outgrowth from primary organoids to passage 20. Scale bar, 100 µm. (E) The cell number of hMGOs during passage 0–3. (F) Western blot analysis of phospho-ERK for mMGOs organoids and hMGOs cultured under the indicated treatment. β-Actin served as a loading control. The relative band intensity that was normalized to the loading control was marked under each band. *n* = 2 biological replicates. (G) Schematic diagram of hMGOs orthotopic transplantation in NSG mice. (H) Representative image of hMGOs orthotopic transplantation. Black dashed box indicates the region of the xenograft. (I) Immunofluorescence staining for human NuMA and LipidTox staining of human engrafted organoids. Lower panel: higher magnifications of the upper panel. Scale bar, 50 µm. (J and K) Immunofluorescence staining for KRT1, PPARγ (J), human NuMA, human KRT14 (K), ZO-1, and P63 (L) in engrafted MG organoids. Scale bar: 50 µm. See also Figs. S6 and S7.

Next, we attempted to establish human MG organoids (hMGO). Refer to the murine organoid, we also mapped the expression pattern of FGFR2, LGR5, and NAMPT in human MG tissue (Fig. 4A). Unexpectedly, NAMPT was barely expressed in human MG tissue (Fig. 4B), so we suspected that its substance, nicotinamide, might not be required for the human organoid culture. Indeed, the mMGO expansion medium could not support hMGO expansion for more than 5 days. However, once nicotinamide was removed, the hMGO dramatically expanded, so we defined this formula as hMGO expansion medium (Fig. 4C). As expected, the hMGO medium could support long-term expansion of hMGO to 20 passages (P20), as well as nearly 10 times cell amplification per passage (Fig. 4D and 4E).

To further confirm the inhibitory effect of nicotinamide on hMGO growth, we added nicotinamide back to hMGO medium and found it significantly suppressed cell proliferation and lipid synthesis (Fig. S6A–E). Bulk RNA-seq revealed that nicotinamide supplement downregulated cell proliferation and lipid-producing-related genes, including *PCNA*, *MKI67*, *PPARG*, and *SCD* (Fig. S6F). Gene ontology (GO) term and Gene set enrichment analysis (GSEA) analysis further confirmed that nicotinamide inhibited cell proliferation and PPAR-related signaling pathways (Fig. S6G and S6H). Unexpectedly, nicotinamide also downregulated FGF10–FGFR2 signaling (Fig. S6H), implying the crosstalk between nicotinamide and FGF10 signaling. To further explore the mechanism, we investigated the phosphorylated ERK (pERK) level in organoids upon FGF10 and nicotinamide treatment. Different from mMGO, whose pERK level was not altered by nicotinamide, the hMGO’s pERK was quite sensitive to nicotinamide and FGF10: FGF10 treatment significantly upregulated pERK, which was suppressed by nicotinamide subsequently (Fig. 4F). To ensure the necessity of other gradients in hMGO medium, we subtracted each component and evaluated the organoid growth, and confirmed the current hMGO medium is the minimal formula (Figs. S6I–K, S7A and S7B). The hMGO cultured with this medium also preserved the key makers (KRTs and PPARγ) and lipid synthesis capacity, compared with parental tissues (Fig. S7C and S7D).

Dry eye syndrome caused by MGD is a chronic disease that cannot be completely cured theoretically, so we are wondering if the transplanted hMGO could generate functional MGs. We transplanted hMGO into the MG sites of immunodeficient nonobese diabetic (NOD) severe combined immunodeficiency (SCID) gamma (NSG) mice (Fig. 4G). After 1 month, the recipient MGs were collected for analysis. The injected organoids could be observed in the MG tissue (Fig. 4H). The human-derived organoids were confirmed by the staining of human NuMA and KRT14 in the engraftment organoids (Fig. 4I and 4K). Through LipidTOX detecting, the secreted lipids were detected in the center of the engrafted organoids (Fig. 4I). Furthermore, a similar level of lipid production was observed in the engrafted organoids and the host MGs, confirming that the engrafted organoids acquired functional maturity *in vivo* (Fig. 4I). Additionally, the engrafted cells retained the expression of functional MG biomarkers, including KRT1, KRT14, PPARγ, and P63α, corresponding with the host tissues (Fig. 4J and 4K). Taken together, the long-term expandable hMGOs can be engrafted into mice and exhibit features of functional maturation, including lipid production and lineage-specific marker expression.

### Dissection of single-cell atlas in human MGs and organoids

**Figure 5.**
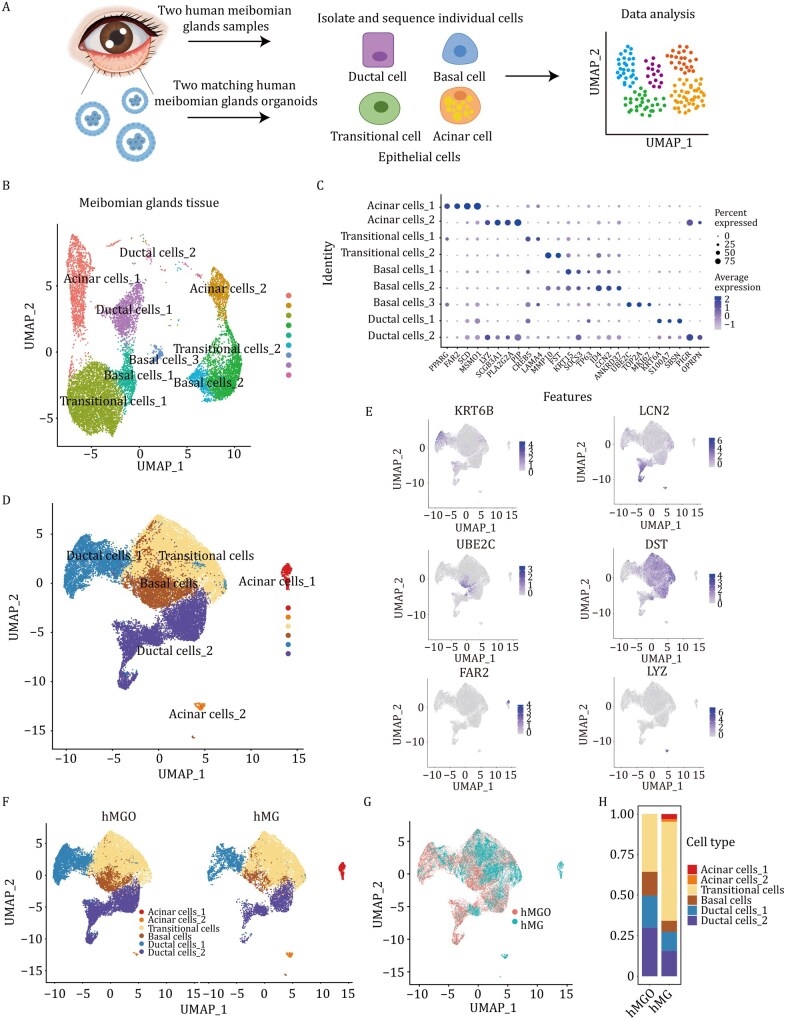
**Mapping the single-cell atlas for human MG tissue and organoids**. (A) The schematic diagram of single-cell mRNA sequencing for 2 human MG tissue samples and matching organoids. (B) UMAP representation of 9 clusters of human MG epithelial cells (*n* = 12,707). (C) The expression level of MG cell markers and cluster-specific genes in different clusters of human MG tissue. (D) Cell clusters were visualized for h hMGOs and tissue cells. (E) The expression of cluster-specific genes in organoids and tissue cells. (F) UMAP plot showing that the parallel cell clusters were identified in organoid and tissue cells. (G) UMAP of human MG cells from organoids (tomato dots) and tissue (cyan dots). (H) The proportion of diverse cell types in hMGOs and tissue. See also Fig. S8.

To dissect the cellular composition and heterogeneity in human MGs and organoids, we performed parallel single-cell RNA sequencing (scRNA-seq). The human MG tissues were digested into single cells and subjected to 10× genomics-based scRNA-seq platform (Fig. 5A). After quality control, a total of 23,323 cells were obtained for further analysis, and six cell types were identified (Fig. S8A and S8B). Focusing on the epithelial homeostasis, we selected *KRT14*^+^ MG epithelial cells (*n* = 13,684) and further identified nine subclusters according to the established cell markers (Fig. 5B and S8C–G). Based on the high expression of the mature lipid-synthetic marker genes, we identified two types of differentiated acinar cells. Type 1 acinar cells (*FAR2^+^*) are featured by lipid metabolic genes, including *PPARG*, *SCD*, *FAR2*, and *MSMO1*. While type 2 acinar cells (*LYZ^+^*) are featured by secretive genes, including *LYZ*, *SCGB3A1*, *PLA2GA2*, and *PIP*. Thus, those two types of acinar cells were identified as mature acinar cells (Fig. 5C and S8G). We also identified other cell lineages of MGs, including MG basal cells (*KRT15*^+^/*SOCS3*^+^; *ID4*^+^/*CCN4*^+^; *UBE2C*^+^/*TOP2A*^+^), transitional cells (*CREB5*^+^/*LAMA4*^+^; *MMP10*^+^/*FST*^+^), and ductal cells (*PIGR*^+^/*OPRPN*^+^; *KRT6A*^+^/*SBSN*^+^; Fig. S8D–F).

In parallel, we also sequenced 22,077 cells in MGOs. After aligning with human MG tissues (*KRT14*^+^), the total cells could be aligned and regrouped into five different clusters, namely acinar cells (type 1 and 2), ductal cells (type 1 and 2), transitional cells, and basal cells (Fig. 5D–G). Notably, the type 1 acinar cells (*FAR2*^+^) were absent in the organoids, while another type of acinar cells (*LYZ*^+^) had a much lower abundance in the organoids (Fig. 5E–G). This indicated that hMGOs retained a relatively immature status. Besides, the cell component proportion analysis also revealed an increase in the ductal cells and basal cells in organoids and a decrease in transitional cells (Fig. 5H), indicating hMGOs preserved more stemness-related cells than tissue. Collectively, the conducted single-cell atlas described parallel heterogeneous cell types between the organoids and MGs, and pinpointed the maturation gap between the organoid and tissue.

### Human MG organoids generated featured lipidome

**Figure 6.**
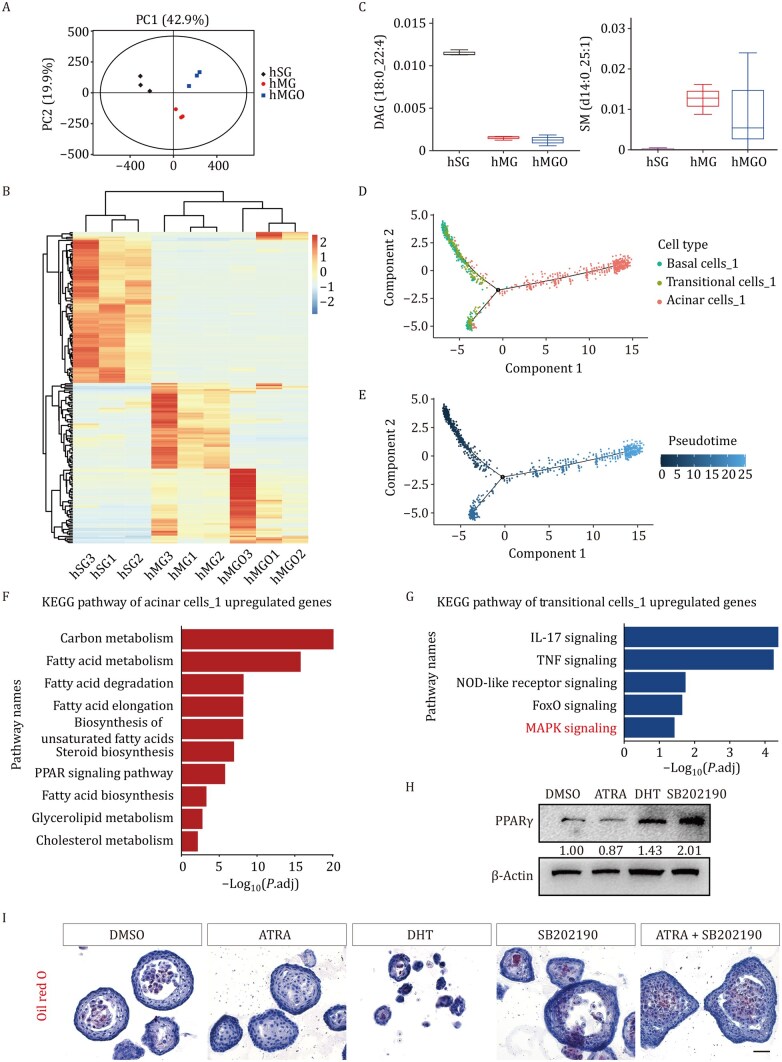
**MAPK signaling inhibition promotes the maturation of hMGOs**. (A) PCA plot of human sebaceous gland (hSG), meibomian gland (hMG), and meibomian gland organoids (hMGO) in lipidomics analysis. 3 independent donors in each group. (B) Heatmap of differential lipid species among hSG, hMG, and hMGO. (C) Relative value of DAG (18:0_22:4) and SM (d14:0_25:1) in hSG, hMG, and hMGO. (D) Position of the MG cells in different cell clusters along the pseudotemporal trajectory. (E) Ordering of MG tissue cells along a pseudotemporal trajectory. MG cells are colored by pseudotime. (F and G) KEGG analysis of upregulated genes (F) and downregulated genes (G) that were differentially expressed in MG acinar cells, compared with MG transitional cells. (H) Western blot analysis of PPARγ for hMGOs cultured under the indicated treatments: 1% DMSO, 100 µmol/L ATRA, 10 µmol/L DHT, and 0.5 µmol/L SB202190. β-Actin served as a loading control. The relative band intensity that was normalized to the loading control was marked under each band. *n* = 2 biological replicates. (I) The Oil Red O staining of hMGOs cultured under the treatment of ATRA, DHT, and SB202190. Scale bar: 50 µm. See also Figs. S9 and S10.

Since we have proved that hMGOs could reproduce the capacity of lipid synthesis (Fig. S7D), we wonder whether the lipid contents of organoids would resemble those of tissues. In order to depict a broader landscape of lipidome, we initially performed non-targeting lipidomic analysis: using human SGs as control, the lipid samples of human MGs and organoids were extracted and coupled with an electrospray ionization high-resolution mass spectrum (MS) analysis. Then, the normalized intensity profiles of the identified lipid compounds were generated. On principal component analysis (PCA) score plot, MGs and organoids are distinctly separated from the SGs sample (Fig. 6A), and hierarchical clustering grouped MG tissue and organoids together, confirming organoids shared a similar lipidome with MG tissue (Fig. 6B). Then, we probed the content of subclass lipids in hMGOs normal MG tissue, and SG tissue. Distinguished with SG, MG tissue and organoids shared a similar level of sphingomyelins (SM), phosphatidylglycerols (PG), and phosphatidylserines (PS). While diacylglycerols (DAG), the lipid content mainly secreted in SG, were much lower in MG tissue and organoids (Figs. 6C, S9A and S9B). Apart from the similarities, we also noticed the 240 differential lipids between hMG and hMGO (Fig. S9C). Among those, FAHFA and TAG, which were detected in human meibum (Butovich et al., 2021; Riecan et al., 2022), were downregulated in hMGO (Fig. S9D).

In summary, consistent with scRNA-seq data above, the lipidomic comparison between MG tissue and organoids not only displayed the overall consistency, but also suggested the maturation gaps, such as terminal differentiated cell types and featured lipids.

### MAPK signaling inhibition boosted human MG organoids’ maturation

Since the hMGOs are less mature than MG tissues, we reasoned that the underlying signals in MG tissues could enhance organoid maturation. To unveil the mechanism of acinar cell maturation, lineage trajectory analysis was performed based on the scRNA-seq data of MG tissues. On the pseudotime plot, basal cells, transitional cells, and acinar cells of MGs are distributed along the pseudotime trajectory. The type 1 acinar cells (*FAR2*^+^), which were identified as the mature MG cells, were located at the rightmost of the trajectory (Fig. 6D and 6E), suggesting that the differentiation path of basal cells–transitional cells–acinar cells. Then we analyzed the signaling pathway enriched in acinar and transitional cells, in order to find key signaling pathways guiding acinar cell maturation. In acinar cells, multiple lipid synthesis-related signaling pathways were activated (Fig. 6F), while the main signaling pathways enriched in transitional cells were inflammation-related pathways such as the IL-17, TNF, NOD-like receptor (NLR), FoxO, and MAPK signaling pathways (Fig. 6G). Interestingly, MGD patients were generally associated with increased tear cytokines, such as TNF-α, IL-17A, and IL-1β (Yu et al., 2022). Reducing the inflammatory cytokines could relieve both the signs and the symptoms of MGD (Li et al., 2021; Yu et al., 2022). Thus, we exposed hMGOs to the inhibitors of the IL-17, TNF, NLR, FOX, and MAPK signaling pathways and attempted to enhance the lipid production capacity of hMGOs. Notably, a selective p38MAPK signaling pathway inhibitor, SB202190, showed the potential to facilitate MGO maturation.

As previously reported, the p38MAPK signaling pathway contributes to cell apoptosis and the autophagy of adipogenic cells and plays a crucial role in inflammation-induced MGD (Liu et al., 2022; Sui et al., 2014). Treatment with SB202190 could significantly upregulate the expression of PPARγ in organoids, and it is more effective than DHT in this regard (Fig. 6H). Using Oil Red O staining, we observed that upon SB202190 stimulation, the produced lipids, which were originally in the cytoplasm, were released into the cavity of the organoids (Fig. 6I). Although the ATRA treatment decreased lipid secretion, SB202190 mitigated the inhibiting effect of ATRA (Fig. 6I). These results confirmed that SB202190, a selective inhibitor of the p38MAPK signaling pathway, could induce the maturation of MGOs.

The mature meibum is featured by ultra-long chain lipids with fatty acid and/or fatty alcohol chains up to C34–C36, including cholesteryl esters (CE), (O)-acylated ω-hydroxy fatty acids (OAHFA), and wax esters (WE) (Butovich, 2017; Butovich and Wilkerson, 2022). However, the non-targeting lipidomic analysis could not robustly detect those major components, especially for the WE. To depict the maturity of human meibomian organoids, targeting lipidomic analysis was performed to evaluate the content of key lipids of meibum, including CE, OAHFA, and WE. Overall, hMGO resembled the lipidomic features of human MG tissue (hMG) in the PCA plot, compared with human periocular skin tissue (hST) (Fig. S10A). Then, we used heatmaps to delineate the similarity between hMG and hMGO on CE, OAHFA, and WE. Compared with the negative control hST, the average content of those featured lipids in hMG and hMGO is higher than hST (Fig. S10B–D). However, we also observed the variant lipidomic performance of hMGO, which showed less mature phenotype (such as hMGO1), suggesting the improving space for hMGO culture system.

## Discussion

Efforts have been made to establish a 3D meibomian culture model, including a slice culture model and explant culture methods (Rötzer et al., 2019; Wang et al., 2023; Xu et al., 2020; Zahn et al., 2022, 2025). Although these models preserved the tissue architectures and functions, such as lipid production, they could not sustain for more than 7 days *in vitro*. In this study, we achieved long-term expansion of murine and human MGOs, which retained all cell lineages and the capacity for lipid synthesis. Thus, the MGOs enable precise drug response and functional engraftment. In addition, MGOs also assist in untangling the underlying mechanism of tissue homeostasis and dysfunction. We found that FGF10 signaling is crucial for organoid expansion and MGs regeneration; NAMPT was barely expressed in human MG tissue, and nicotinamide specifically hindered hMGOs expansion, indicating the distinct NAD metabolism between mouse and human; Single-cell atlas of human MG epithelial cells indicated MAPK signaling inhibition is required for acinar cell differentiation, thus treated with p38MAPK signaling pathway inhibitor, SB202190, promote organoid maturation.

MGD is the leading cause of dry eye disease. Clinically, the main treatments for MGD include cleaning the eyelids through hot compresses and massages, instilling artificial tears containing lipid components, and using antibiotic drugs to reduce inflammation. Currently, the development of artificial tears is prevailing. In order to make the artificial tears as similar as possible to the composition of tears, various substances are added, including mineral oil and phospholipids (Jones et al., 2017). We identified that FGF10 supported both murine and human MGO expansion. And FGF10 eye drops also rescued the ATRA-induced MGD (Figs. 1 and 2). Our data suggested that the FGF10 signaling is not only required for MG development and homeostasis (Reneker et al., 2017; Tao et al., 2005; Tsau et al., 2011), but also prevents the progression of MGD. Therefore, FGF10 eye drops are a promising reagent for human dry eye disease, which needs more human-relevant tests for its application. In addition to those physiological restoration, FGF10 treatment also increased the proliferation of acinar cells, instead of ductal cells (Fig. 2N). Consistent with our observation, FGFR2 deletion severely affected the proliferation and differentiation of MG acinar cells but affected MG ductal cells to a lesser extent (Reneker et al., 2017). We also found that the proliferating cells in acini are mainly located at the edges (Fig. S3A), which were likely basal cells. Consistent with these data, the MGOs also showed proliferative Krt5^+^ basal cells under the FGF10-containing medium (Fig. 1E). Hence, it is conceived that basal cells are the major proliferative cells that respond to FGF10 signals.

NAMPT generates nicotinamide mononucleotide from nicotinamide and 5′-phosphoribosyl-1-pyrophosphate, thereby catalyzing the rate-limiting step in the mammalian NAD salvage pathway (Garten et al., 2015). Therefore, the bonus of nicotinamide largely depends on the activity of NAMPT. We found the distinct expression level of NAMPT in MGs between mouse and human (Fig. 4B). And the addition of nicotinamide into human organoid culture medium suppressed the organoid growth (Fig. 4C). Previous studies also revealed that high doses of nicotinamide may exert adverse effects, such as DNA damage, increased intracellular ROS, spindle defects, and mitochondria dysfunction (Oikawa et al., 1980; Utakoji et al., 1979; Zhang et al., 2016). In this study, we found that nicotinamide inhibited the activity of FGF10 signaling through decreasing the pERK level in human organoids. But how nicotinamide crosstalk with the upstream of FGF10 signaling need further investigation. It was reported that the prevalence of dry eye disease was higher in female than male (Kim et al., 2023). Considering that nicotinamide is frequently used in cosmetics, it’s likely that excess nicotinamide exposure is also an emerging risk factor for MGD.

MAPK signaling participates in various cellular programs like proliferation, differentiation, transformation, and apoptosis (Zhang and Liu, 2002). Leveraging scRNA-seq analysis, we found that the MAPK signaling pathway was significantly enriched in transitional cells (immature acinar cells), compared to mature acinar cells (Fig. 6G). And identified SB202190, a selective p38MAPK inhibitor, promoted lipid production in MGOs (Fig. 6I), suggesting that MAPK signaling pathway inhibition is required for the differentiation of mature acinar cells. Similar to adipocytes, the p38MAPK signaling was more active in preadipocytes than adipocytes (Aouadi et al., 2006). Consistently, another selective p38MAPK inhibitor, SB203580, could block IL-1β-induced MGD and restore its functions in rats (Qu et al., 2023). These findings indicate that MAPK signaling inhibition enhances the functions of terminally differentiated cells and could be considered as potential targets for MGD. However, whether MAPK inhibition affects the self-renewal of basal cells remains to be further investigated.

MGOs provide the opportunity for cell therapy for MG atrophy. Given that MG atrophy is the terminal stage of MGD, when MG stem cells cannot regenerate the whole MGs, the orthotopic transplantation might be the only choice for those patients. Successfully engrafting MGOs is the first step for potential regenerative therapies (Bannier-Hélaouët et al., 2021). The established MGO culture and differentiation protocol would provide the “seed cells” for bioengineered MG grafts. Efforts should be made to improve the orthotopic transplantation efficiency and reduce the immune rejection effects caused by xenotransplantation. In conclusion, we established functional, long-term cultured MGO cultures for MG homeostasis and diseases research, which provides a useful platform for the development of associated medical interventions and regenerative medicines.

In MGs, the acini are connected to the main duct by small ducts, so that the lipid produced by the meibocytes can be transported through the ducts to the eyelid. Although our organoid model contained all cell types of MGs, the ductal cells resided with basal cells to form the outer layer of the organoid, losing their function to drain out the meibum. Additional optimization of MGO establishment is required to develop ducts-acinus structure.

In addition, we showed that the hMGOs engrafted upon orthotopic transplantation, and the transplanted organoids could produce comparable lipids to their neighboring tissue. However, whether the MGO transplantation could rescue the MG dysfunction remains to be further investigated.

## Supplementary Material

pwaf095_Supplementary_Data

## Data Availability

Single-cell and bulk RNA-seq data have been deposited at the NCBI SRA database and are publicly available with the accession number: SRP497138. Lipidomics raw data have been deposited to the MetaboLights with the accession number: MTBLS9799, and can be fast downloaded from Google Drive with the link: drive.google.com/drive/folders/1mb84LM6ZEaX-naEBJ24hQx9nLBJ7K7Ix? usp=sharing. Any additional information required to reanalyze the data reported is available from the lead contact upon request.

## Code availability

This paper does not report original code.
